# Daily practices in chemotherapy for advanced gastric or gastroesophageal junction adenocarcinoma: METESTOMAC French prospective cohort

**DOI:** 10.1002/cam4.5354

**Published:** 2022-11-16

**Authors:** Sylvain Manfredi, Marie Dior, Olivier Bouche, Emilie Barbier, Vincent Hautefeuille, Marielle Guillet, Justine Turpin, Vincent Bourgeois, Dall Osto Helene, Romain Desgrippes, Franck Audemar, Yann Molin, Christophe Locher, Thierry Chatellier, Thierry Lecomte, Nathalie Baize, Cedric Lecaille, Dominique Spaeth, Gael Goujon, Come Lepage, David Tougeron, For The Metestomac Investigators/Collaborators

**Affiliations:** ^1^ Digestive Cancer Registry of Burgundy, INSERM, LNC UMR1231 University Bourgogne Franche‐Comté, Dijon‐Bourgogne University Hospital Dijon France; ^2^ University Hospital Cochin Paris France; ^3^ University Hospital R Debre Reims France; ^4^ CRGA, FFCD, INSERM, LNC UMR1231 University Bourgogne Franche‐Comté Dijon France; ^5^ University Hospital Amiens Picardie Amiens France; ^6^ University Hospital Croix Rousse Lyon France; ^7^ General Hospital Abbeville Abbeville France; ^8^ General Hospital Duchenne Boulogne sur mer France; ^9^ Private Hospital Forcilles Ferolles Attilly France; ^10^ General Hospital Saint Malo France; ^11^ General Hospital Côte Basque Bayonne France; ^12^ Private Hospital La Sauvegarde Lyon France; ^13^ General Hospital Meaux France; ^14^ Private Hospital Clinique mutualiste de l'estuaire Saint Nazaire France; ^15^ University Hospital Tours Tours France; ^16^ University Hospital Angers Angers France; ^17^ Private Hospital Bordeaux Nord Bordeaux France; ^18^ Private Hospital Gentilly Nancy France; ^19^ University Hospital Bichat Paris France; ^20^ CRGA, FFCD, INSERM, LNC UMR1231 University ‘Bourgogne Franche‐Comté’ Dijon France; ^21^ University Hospital La Miletrie Poitiers France

**Keywords:** cohort, gastric cancer, real life, strategy

## Abstract

**Background:**

Around 50% of gastric cancers are diagnosed at an advanced stage. Several chemotherapy regimens are now internationally validated. Few data are available on the routine daily management of advanced gastric or gastroesophageal junction cancers. We aimed to describe chemotherapy practices, tolerance, and efficacy overall survival (OS) and Progression free survival (PFS) in a prospective French cohort.

**Methods:**

Patients starting palliative chemotherapy were prospectively enrolled in 49 French centres. The primary objective was to report and describe patients' characteristics and treatment strategies. Secondary objectives were OS, PFS, objective response rate, adverse events rate, performance status deterioration during the chemotherapy.

**Results:**

A total of 182 patients were included; 179 were analysed. Most patients received platinium‐based chemotherapy as the first treatment and FOLFIRI as second; 62.0% of patients received a second line, and 32.4% a third line. More than two thirds of Her2‐positive patients were first treated with trastuzumab. The FOLFIRI regimen was the most frequently used second‐line therapy. Median OS was 13.3 months, similar whatever the chemotherapy or combinations used in the first line. One‐ and 2‐year OS increased with the number of chemotherapy lines received, from respectively 24.7% and 5.7% (1 line), to 46.9% and 12.4% (2 lines) and 88.1% and 29.9% (3 or more lines) (*p* < 0.0001).

**Conclusion:**

Our study showed that treatment strategies in France are based on a succession of doublets, making it possible to offer a second and third line of treatment more often. This treatment strategy must be taken into account for future trials with immunotherapy combinations.

## Graphical abstract

Flow chart of the multicenter prospective METESTOMAC cohort.

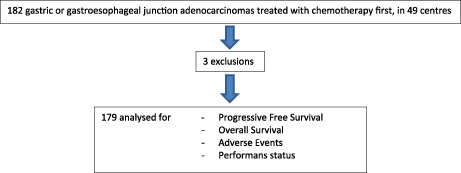

## 1 INTRODUCTION

With 1,034,000 new cases and 783,000 deaths in 2018 worldwide,^1^ gastric cancer (GC) remains a public health problem. In France, despite decreases in incidence and mortality since 1990, gastric and gastroesophageal junction (GEJ) cancers ranked 8th for men and 14th for women, with 6557 new cases (65% males) and 3272 deaths (85% males) in 2018.^2^ Around 50% are diagnosed at an advanced stage and are eligible for chemotherapy as the first‐line treatment.^3^ Despite progress in the management, and a modest increase in 5‐year overall survival (OS) in France from 25.7% (1995–1999) to 27.7% (2005–2009), the prognosis remains poor, particularly in a metastatic setting with a 5‐y OS less than 4%.^4^, ^5^, ^6^

Several chemotherapy regimens, with fluoropyrimidine used alone (FU), or in doublet combinations with platinium or eprubicin or irinotecan, or in triplet combinations with platinium and epirubicin or docetaxel,^7^ are now internationally validated for first and second‐line therapy in patients in general condition Eastern Cooperative Oncology Group (ECOG) 0 or 1 (US (ASCO), European (ESMO), French (TNCD) guidelines). The durations of each line of chemotherapy are not pre‐defined in advance; the chemotherapy protocol is changed when the cancer progresses or for intolerance for patients whose general condition allows chemotherapy to be continued. In patients whose tumour overexpresses the HER2 receptor, the combination of FU‐cisplatin‐trastuzumab as compared with chemotherapy alone (FU‐cisplatin) increased survival.^8^ ECF (epirubicin‐cisplatin‐FU) is an earlier reference scheme.^9^, ^10^ Data from the REAL 3 study^11^ showed equivalence between cisplatin and oxaliplatin, and between FU and capecitabine. ECX, EOF and EOX regimens can replace the ECF scheme. The docetaxel‐cisplatin‐FU (DCF) combination compared to doublet (Fluoropyrimidine plus platinum), increased OS but induced higher toxicity.^12^ Split administration of docetaxel reduced haematological toxicity.^13^ The combination LV5FU2‐cisplatin^14^ or capecitabine‐cisplatin^15^ is widely used.^16^ In a phase III study, FOLFOX showed efficacy equivalent to that of FU‐cisplatin, with superiority of the FOLFOX arm in terms of response rate, time to treatment failure (TTF) and Progression free survival (PFS) in patients over 65 years of age.^17^ FOLFIRI, evaluated in a phase II randomised trial, was better tolerated than LV5FU2‐cisplatin, and provided higher response rates, PFS and OS.^18^ The phase III randomised trial, which compared the first and second‐line sequences OLFIRI‐ECX and ECX‐FOLFIRI, showed that the TTF was greater with FOLFIRI as the first‐line.^19^ Regarding second‐line therapy, irinotecan monotherapy, as compared with best supportive care alone, led to significantly longer OS.^20^ Docetaxel monotherapy at 75 mg/m^2^ as a second line was evaluated against supportive care in a phase III study^21^ and resulted in significantly better OS. In an international randomised study, Ramucirumab monotherapy in the second metastatic line and assessed against placebo was shown to improve OS.^22^ This was also the case when it was used in combination with paclitaxel in a phase III randomised study versus paclitaxel monotherapy.^23^ Although Ramucirumab has obtained European market authorisation, is not reimbursed in France. Despite encouraging results,^24^ continuing trastuzumab beyond progression for Her2‐positive tumours does not seem to be effective.^25^

The choice of the chemotherapy scheme depends on age, WHO performance status, comorbidities, Her2 status and market authorisation. Patients treated in routine practice, are not selected and few data on the routine daily management of advanced gastric or gastroesophageal junction cancers are available. In the METESTOMAC study, we aimed to describe chemotherapy practices, tolerance and efficacy results (PFS and OS) in a prospective French cohort. This study provides data on daily practices beyond the first‐line of chemotherapy and could help physicians to design future treatment strategy trials.

## 2 MATERIALS AND METHODS

### 2.1 Study design

Forty‐nine centres of the FFCD (French Federation of Digestive Cancer) network prospectively registered locally advanced or metastatic gastric or GEJ adenocarcinoma patients treated with chemotherapy between March 22, 2016 and April 28, 2017. The FFCD is a national digestive oncology network that develops and conducts Phase 2 and 3 therapeutic trials and prospective cohorts throughout the territory in partnership with cancer care structures (public hospitals, center for the fight against cancer, private hospitals).This cohort was initially expected to register around 150 patients and finally registered 182 cases. Patients were included at the time of their first‐line chemotherapy and followed for at least 2 years.

### 2.2 Methods

Patients' baseline characteristics included age, sex, TNM stage, previous anti‐cancer treatment (including resection of the primary) in case of recurrence, location of the primary tumour, location of metastases, Her2 expression, ECOG status, and Lauren classification.

Information on chemotherapy was collected prospectively. Chemotherapy regimens were grouped as follows: platinium‐based chemotherapy (oxaliplatin, cisplatin or carboplatin with FU); anthracycline‐based chemotherapy (including ECF, ECX, EOF and EOX schemes: epirubicin, cisplatin/oxaliplatin, FU); taxane‐based chemotherapy (including associations of docetaxel/paclitaxel with FU and cisplatin/oxaliplatin); chemotherapy combined with trastuzumab (including cisplatin/carboplatin/oxaliplatin, FU associations); irinotecan‐based chemotherapy (FOLFIRI or irinotecan alone) and other chemotherapy regimens used as monotherapy, FOLFIRINOX and experimental regimens.

ECOG status at the start of each line of chemotherapy, the number of courses and duration of each chemotherapy regimen, reasons for stopping treatment (progression, toxicity, other), the expected Grade 3 and more adverse events, best response according to RECIST 1.1 criteria were also collected.

The primary objective was to report and describe patients' characteristics and treatment strategies. Secondary objectives were OS, PFS, objective response rate (ORR), adverse events rate, performance status deterioration during the first‐line and further lines of chemotherapy.

### 2.3 Statistical analysis

Descriptive analyses were done for patients' baseline characteristics. Quantitative variables were described with means or medians, standard deviations (SD) or interquartile ranges (IQR) and were compared with the Wilcoxon rank‐sum test. Qualitative variables were described as frequencies and percentages and were compared using the chi‐square test or Fisher's exact test.

OS and PFS curves were plotted using the Kaplan–Meier method and described using medians with two‐sided 95% confidence intervals (95% CI). Log rank tests were used to compare rates and event‐time distributions with a 95% CI.

OS was defined as the time between the start of the first‐line chemotherapy and death (any cause). Alive patients were censored at the date of the last news. PFS was defined as the time between the start of the first‐line chemotherapy and the first progression or death. Patients alive without progression were censored at the date of the last news. The median follow‐up was evaluated using the reverse Kaplan–Meier method. All statistical analyses were done using SAS software 9.4 (SAS Institute).

## 3 RESULTS

### 3.1 Population characteristics

A total of 182 patients were included prospectively from March 2016 to April 2017. Three patients were excluded from the analyses: one for associated lung cancer, one for squamous cell oesophageal cancer and one for a non‐metastatic gastric adenocarcinoma. The 179 remaining patients were included in the analysis (Table 1).

**TABLE 1:** Population characteristics (*N* = 179)

	Mean	SD
Age	65.0	12.8
Male	65.2	11.7
Female	64.2	15.7
	*n*	%
Sex		
Male	133	74.3
Female	46	25.7
Location		
Gastric	118	65.9
GEJ	59	33.0
Unknown	2	1.1
Histology^a^		
Diffuse	80	44.7
Intestinal	75	41.9
Unknown	24	13.4
HER2 status		
Positive	41	22.9
Negative	125	69.8
Unknown	13	7.3
Metastasis		
Synchronous	128	71.5
Metachronous	48	26.8
Unknown	3	1.7

Abbreviations: GEJ, gastroesophageal junction; SD: Standard deviation.

^a^ Lauren classification.

The mean age of patients was 65.0 years (SD 12.8), 74.3% were male, two thirds had gastric cancer (GC) and one third gastroesophageal junction (GEJ) cancer. According to the Lauren classification, 44.7% were of the diffuse type, 41.9% of the intestinal type and 13.4% were unspecified. Metastases were synchronous in 71.5% of cases. The primary tumour was treated in 24.0% of cases (*n* = 43): in seven cases (16.3%) by surgery first, in 36 cases (83.7%) with chemo or chemo‐radiotherapy first, followed by surgery in 26 cases (72.2% of them). The Her2 status was known in 92.7% of cases and positive in 24.7%. The median durations of each line of treatment and each protocol are specified in Table 2.

**TABLE 2:** mean duration of chemotherapy lines and response

	*n*	Median number of courses	[IQR]	Median duration, months	[IQR]	Objective response rate	Stable disease rate
1st line							
Total	178^a^	8.0	[4.0–12.0]	4.2	[2.3–6.2]	34.5	26.6
Platinium	103	8.0	[4.0–12.0]	4.9	[1.7–6.2]	33.3	25.5
Trastuzumab	28	7.0	[6.0–11.0]	4.4	[3.0–6.2]	50.0	17.9
Taxane	19	10.0	[3.0–12.0]	3.8	[1.6–5.6]	42.1	26.3
FOLFIRI	19	9.0	[6.0–14.0]	4.3	[2.3–7.6]	10.5	42.1
Anthracycline	6	4.5	[3.0–6.0]	3.3	[2.1–4.4]	33.3	33.3
Others	3	6.0	[4.0–8.0]	3.3	[1.4–3.4]	0.0	33.3
2nd line							
Total	111	5.5	[3.0–7.5]	2.5	[1.4–5.0]	12.4	24.8
Platinium	20	6.0	[3.0–7.5]	2.4	[1.0–3.8]	17.7	41.2
Trastuzumab	10	5.5	[4.0–8.0]	2.4	[1.6–6.2]	0.0	10.0
Taxane	16	3.0	[1.0–4.0]	1.5	[0.5–2.8]	6.7	13.3
FOLFIRI	50	6.0	[4.0–10.5]	3.3	[1.7–5.7]	16.7	27.1
Ramucirumab	5	3.0	[1.0–6.0]	1.5	[0.5–4.6]	0.0	20.0
Others	10	2.0	[2.0–6.0]	1.8	[0.5–2.4]	10.0	20.0
3rd line							
Total	58	4.0	[2.0–6.0]	2.3	[1.4–4.2]	6.9	19.0
Platinium	7	4.0	[3.0–6.0]	2.1	[1.0–3.0]	0.0	0.0
Trastuzumab	3	9.5	[4.0–15.0]	4.7	[1.5–7.8]	0.0	0.0
Taxane	22	3.0	[2.0–4.0]	2.3	[1.4–3.5]	4.6	18.2
FOLFIRI	19	5.0	[2.0–11.0]	2.3	[1.1–4.9]	10.5	21.1
Ramucirumab	3	6.0	[3.0–12.0]	5.4	[2.3–5.9]	0.0	0.0
Others	5	6.0	[4.0–6.0]	5.1	[4.6–5.4]	20.0	60.0

^a^ Data were missing for only 1 of the 179 patients.

### 3.2 First‐line chemotherapy

Most patients were given platinium‐based chemotherapy (Table 2): 57.9%, (91.3% with Folfox) and an additional 15.7% with a platinium‐based chemotherapy + trastuzumab. Taxane (15.8% doublet, 84.2% triplet; 56% paclitaxel, 44% docetaxel) and irinotecan (FOLFIRI) were each used in 10.7% of cases. Anthracyclines were used in only 3.4% of cases and three patients were treated with other chemotherapies (FOLFIRINOX, 5FU alone, experimental anticancer therapy). More than two thirds (68.3%) of Her2 positive patients were treated with trastuzumab.

**FIGURE 1:**
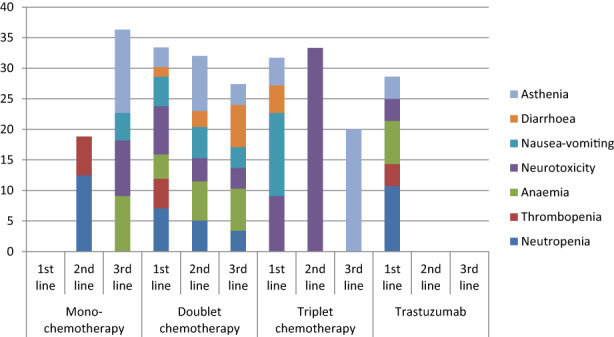
AEs grade ≥3 according to chemotherapy scheme (%)

**FIGURE 2:**
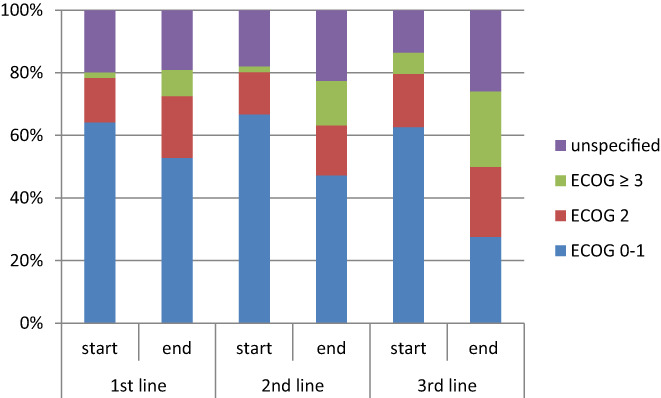
ECOG status evolution for each chemotherapy line

During the first‐line therapy, an objective response (OR) (complete response (CR), or partial response (PR)) was observed in 34.5% of cases and stabilisation in 26.6% of cases. Grade 3 or more toxicity was observed in 33.7% of patients: major biological adverse events were neutropenia (30%), thrombopenia (20%), anaemia (15%); major clinical toxicities were neurotoxicity (19.7%), nausea/vomiting (14.8%), diarrhoea (6.6%), asthenia (4.9%). Details of grade 3 or more toxicity by chemotherapy scheme and line are provided in Figure 1. At the end of the first‐line chemotherapy, 59.1% of patients were considered progressive (radiological progression in 82.7% and clinical in 17.3%). At the start of the first line, the ECOG status was 0–1 in 64.2%, 2 in 14.2%, unspecified in 19.9% and 52.8%, 19.7% and 19.1%, respectively at the end of this line (Figure 2).

### 3.3 Second and subsequent lines of chemotherapy

After the first‐line chemotherapy, 111 patients (62.0%) received a second line (Table 2), 58 (32.4%) a third line, and 26 (14.5%) more than three lines (18 received 4 lines, 7 received 5 lines and 1 received 6 lines).

The FOLFIRI regimen was the most frequently used second‐line therapy in 45% of cases, platinium‐based chemotherapy in 18% of cases, and taxane in 14.4%. Trastuzumab was used in 10 (9%) cases, in continuation of the first line in eight cases (28.6% of patients treated in the first line with trastuzumab continued with trastuzumab beyond progression). Ramucirumab was used in five patients in association with taxane.

Toxicity grade ≥3 was observed in 30.2% of patients; 12 patients had biological toxicity: anaemia, neutropenia and anicteric cholestasis were the most frequent, respectively 29.4%, 17.7% and 17.6% of cases; 30 patients had clinical toxicity: deterioration in general health (anorexia, weight loss, and asthenia) in 24.4%, neuropathy in 15.6%, and nausea‐vomiting in 8.9% (Figure 1). At the end of the second line, 74.8% of patients were considered progressive. At the start of second line, the ECOG status (Figure 2) was 0–1 in 66.7% of patients, 2 in 13.5%, unspecified in 18.0%, and respectively 47.2%, 16.0% and 22.6% at the end of this line.

Taxane and FOLFIRI were the most frequently used third‐line chemotherapy regimens (Table 2), respectively 38.2% and 32.7%. Platinium‐based chemotherapy was used in 12.7% of cases, trastuzumab, ramucirumab and other schemes were each used in 5.5% of cases. Toxicity ≥3 was observed in 31.0% of patients: six patients had biological toxicity: anaemia in 66.7% and neutropenia in 33.3%; 19 patients had clinical toxicity: deterioration in general health (anorexia, weight loss, and asthenia) in 26.1%, nausea‐vomiting in 21.8%, neuropathy in 13.1%, and diarrhoea in 13.0% (Figure 1). At the end of the third line 82.8% of patients were considered progressive. At the start of third line, the ECOG status (Figure 2) was 0–1 in 62.7%, 2 in 17.0%, unspecified in 13.6%, and respectively 27.6%, 22.4% and 25.9% at the end of this line. The most frequently used sequences for the 58 patients who had three lines of chemotherapy were platinium‐irinotecan‐taxane in 24% and platinium‐taxane‐irinotecan in 10%.

### 3.4 Overall survival

**FIGURE 3:**
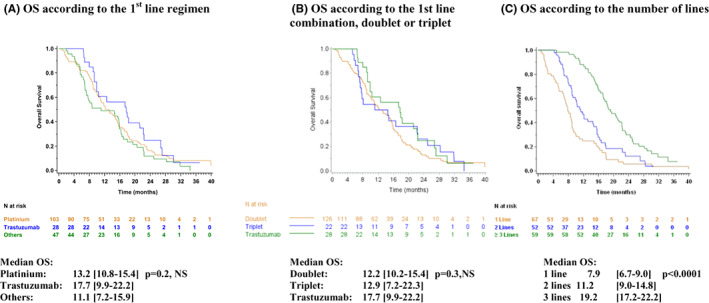
Overall survival (OS)

Median follow‐up was 31.1 months [95% CI: 30.1–39.9]. At the time of the analysis 153 patients (85.5%) had died. The median OS was 13.3 months [95% CI: 10.8–15.6], with a 12‐month survival rate of 53.3% [95% CI: 45.6–60.5], and a 24‐month survival rate of 16.3% [95% CI: 11.0–22.5]. OS was no different regardless of the chemotherapy regimen (*p* = 0.2, NS) (Figure 3A) or combinations (doublet/triplet) used (*p* = 0.3, NS) (Figure 3B) in the first line. One‐ and 2‐year OS increased with the number of chemotherapy lines received, from respectively 24.7 [95% CI:14.5–36.3] and 5.7 [95% CI:1.5–14.1] in patients receiving only one line, to 46.9 [95% CI:32.8–59.9] and 12.4 [95% CI:4.5–24.6] in patients receiving two lines and 88.1 [95% CI:76.7–94.2] and 29.9 [95% CI:18.7–41.8] in patients receiving three or more lines (*p* < 0.0001) (Figure 3C).

Only eight out of 28 patients continued trastuzumab in the second line, which was too small a number to conduct a meaningful analysis of the impact of continuing trastuzumab beyond the first line.

### 3.5 Progression free survival

**FIGURE 4:**
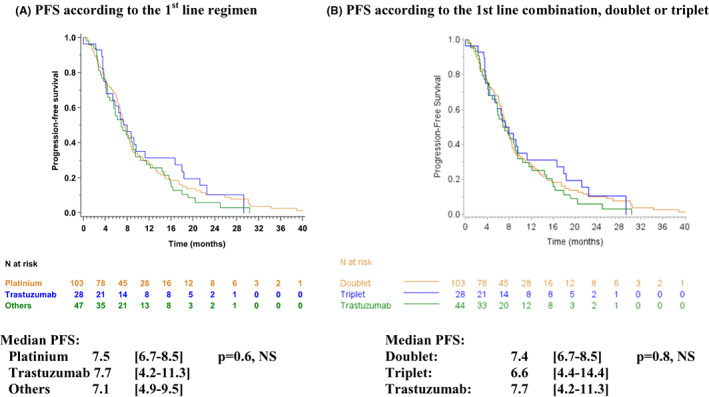
progression free survival (PFS)

At the end of the study, 166 patients (92.7%) had progressed during the first‐line treatment. Median PFS was 7.4 months [95% CI: 6.7–8.4], with a 12‐month PFS of 28.8% [95% CI: 22.3–35.7], and a 24‐month PFS of 9.0% [95% CI: 5.2–17.1]. PFS was no different whatever the chemotherapy (*p* = 0.6, NS) or combinations (doublet/triplet) used in the first line (*p* = 0.8, NS) (Figure 4A,B).

## 4 DISCUSSION

Our national prospective cohort study provides important information on the management in current practice. The distribution between gastric cancer and GEJ cancer in our cohort is similar to that reported by the Russian and South Korean real‐life cohorts^26^, ^27^ and that of randomised trials, which include between 69% and 88% of gastric cancers.^22^, ^25^, ^28^ The HER2 status was determined in almost all of the patients in our cohort, in accordance with international recommendations; it was determined in only 42% and 46% of cases in the Russian and South Korean cohorts, respectively.^26^, ^27^ Cancers were HER2 positive in 23% of patients in our cohort, which is similar to rates in previous studies, ranging from 15% to 33%.

In patients with an ECOG ≤2, the European and French guidelines^29^, ^30^ recommend doublet or triplet first‐line treatment with FU, platinum, taxane or irinotecan for HER2 negative cancer, and with FU, platinum and trastuzumab for HER2 positive cancer. In daily practice, the patients in our cohort mainly received platinum‐based chemotherapy (73.6%), which is more than in the Russian cohort (54%), and similar to that in the South Korean cohort (75%). Trastuzumab was used as the first line, alone or in combination, in 63% of our HER2 positive patients (16% of all patients treated); taxane in doublet or triplet with platinum, and irinotecan were each used in 11% of cases. This distribution of chemotherapy regimens differs from those reported by the Russian and the South Korean cohorts, where FU alone was used in 17% and 19%, respectively, and taxane in 7% of cases in Russia.^26^, ^27^

The use of doublet or triplet therapy with taxane remained low in our cohort, probably due to the fact that these were recent standards at the time.

Sixty‐two percent of the patients in our cohort received a second line of treatment, a higher proportion than in the French strategic trial (39% in the FOLFIRI arm, 48% in the ECX arm),^19^ and higher than in the UK study,^31^ which preferentially used a triplet ECF as the first line, but lower than in the two real‐life cohorts: 80% in South Korea, 86% in Russia, where they preferentially used a mono‐chemotherapy regimen as the first line. Similar results were found for the third line. These differences in exposure to a second and third line are probably due to differences in the selection of patients, an improvement in the management of side effects and the use of the LV5FU2 regimens associated with cisplatin or oxaliplatin in France, and the use of triplet ECF preferred in UK.

French guidelines recommend the use of chemotherapy in the second line, depending on the first‐line treatment: Irinotecan, taxane, ramucirumab alone or in combination (not reimbursed in France). The most widely used second‐line chemotherapy in our study was FOLFIRI (45%), used more often than in the Russian (15%) and South Korean cohorts (19%).^26^, ^27^ Platinum and taxanes were used in 18% and 14% of cases, respectively, as in the Russian cohort (19% and 14%), and more often than in the South Korean cohort (13% and 8%).^26^, ^27^ Trastuzumab was continued as the second line in 28.6% of patients who had it in the first line, even though this was outside the marketing authorization: a randomised phase‐2 study showed the lack of benefit of continuing trastuzumab in the second line after failure of a first line combining 5FU, platinum and trastuzumab,^25^ while retrospective series suggest that maintaining HER2 blockade is feasible^32^, ^33^ and would lead to increased survival.

Although there are no recommendations with a high level of evidence, a third line of treatment, mainly taxane (monotherapy in 79.2%) or FOLFIRI, was given in nearly a third of the patients in our cohort.

The results for OS, PFS, objective response and toxicity in our cohort are comparable to those of the randomised trials that validated the different chemotherapy regimens. The median survival of 13.3 months observed in our study is slightly better than that of 11 months reported in the 2017 Cochrane review,^3^ which reported the results of first‐line chemotherapy regimens. The increase of 3.5 months provided by a second line of chemotherapy observed in our study is slightly lower than that reported by the meta‐analysis, using parametric modelling methods, with an increase of 4.0 to 9.6 months.^34^ The best OS is observed in patients receiving three or more lines of chemotherapy, resulting in a median survival of 19.2 months [95% CI: 17.2–22.2], that is, a gain of 11.3 months compared with patients receiving only one line. These survival medians are comparable to those reported in a large single‐centre study: 8.3 months after one chemotherapy line, 14.0 months after two lines, and 20.1 months after three lines.^31^ The continuation of chemotherapy after the second line in patients who can withstand it, 32.4% of our population (80% ECOG ≤2), appears justified. In our study, OS did not depend on the chemotherapy regimen used in the first line. The most frequently used therapeutic sequence in our study was FOLFOX in the first line for 53% of patients followed by FOLFIRI in the second line for 33% of them and taxane as monotherapy in the third line for 42% of them. The best OS was observed in patients receiving trastuzumab: median OS of 17.7 months [95% CI: 9.9–22.2], which is better than that reported in the TOGA study (median OS: 13.8 months).^8^ This difference in survival also found in another study,^31^ is probably explained by a better selection of HER2 positive patients (only IHC 2+ and positive FISH or IHC 3+ are authorised to receive trastuzumab in France). Our study showed that the treatment strategy in France is based on a succession of doublets, making it possible to offer more often a second and a third line of treatment, perhaps extending patients' overall survival while limiting the side effects and the deterioration in the ECOG status.

Our study has some limitations: First, it was an observational, non‐randomised study with small subgroups, which does not allow direct comparisons of the different treatments; secondly, side effects were not collected as accurately as in a randomised trial and therefore probably over or under recorded. The strength of our study lies in the analysis of chemotherapy practices for metastatic gastric cancer in representative French centres. Our results are comparable to those from randomised trials in this population and reflect compliance with the recommendations and the quality of the multidisciplinary meetings, mandatory in France. The therapeutic sequence the most frequently used in current practice in France is a platinum‐based regimen in the first line and irinotecan in the second line. In France, the ongoing GASTFOX phase III study will compare TFOX versus FOLFOX as the first‐line chemotherapy for patients with advanced gastric or GEJ adenocarcinoma.^35^ Given the recent results of immunotherapy,^28^, ^36^, ^37^ this therapeutic sequence seems to be the most interesting for the development of trials for future strategies that include immunotherapies, depending on the combined positive score, as suggested in an editorial.^38^ Since FOLFIRI is a standard of care after doublet or triplet platimun‐based first line, it is relevant to combine FOLFIRI with immunotherapy as in the DURIGAST trial^39^ recently communicated at the ESMO 2022 congress.^40^ Immunotherapy is now essential in the treatment of gastric adenocarcinoma, it remains to define the best combinations and the best sequences of associated chemotherapy. For the time being, taxanes are probably used primarily in younger fit patients: therapeutic trials dedicated to this population eligible for treatment with taxane could be developed.

## 5 CONCLUSION

This real‐life cohort study confirmed that the results of therapeutic trials, in terms of efficacy and safety, are applied in current daily practices, assessed the proportion of patients receiving a second line or more, corroborated compliance with the recommendations and provided data for the design of future trials.

### 5.1 Ethics and legal considerations

This study did not fall within the scope of the modified biomedical research law known as the Huriet‐Sérusclat law of August 9, 2004. In application of articles 40–1 of the “Informatique et Liberté” law of January 6, 1978 amended by the law of August 9, 2004, the FFCD declared the study to the CCTIRS (*Comité consultatif sur le traitement de l'information en matière de recherche*) and the CNIL (*Commission nationale de l'informatique et des libertés*). In accordance with the law, an information document was given to patients.

## AUTHOR CONTRIBUTIONS

**Sylvain Manfredi:** Conceptualization (lead); formal analysis (equal); funding acquisition (lead); investigation (equal); methodology (lead); writing – original draft (lead); writing – review and editing (lead). **Marie Dior:** Data curation (equal); investigation (equal); writing – review and editing (equal). **Olivier Bouche:** Data curation (equal); investigation (equal); writing – review and editing (equal). **Emilie Barbier:** Data curation (lead); formal analysis (lead); methodology (lead); software (lead); writing – review and editing (equal). **Vincent Hautefeuille:** Data curation (equal); resources (equal); writing – review and editing (equal). **Marielle Guillet:** Data curation (equal); investigation (equal); writing – review and editing (equal). **Justine Turpin:** Data curation (equal); investigation (equal); writing – review and editing (equal). **Vincent Bourgeois:** Data curation (equal); investigation (equal); writing – review and editing (equal). **Dall Osto Helene:** Data curation (equal); investigation (equal); writing – review and editing (equal). **Romain Desgrippes:** Data curation (equal); investigation (equal); writing – review and editing (equal). **Franck Audemar:** Data curation (equal); investigation (equal); writing – review and editing (equal). **Yann Molin:** Data curation (equal); investigation (equal); writing – review and editing (equal). **Christophe Locher:** Data curation (equal); investigation (equal); writing – review and editing (equal). **Thierry Chatellier:** Data curation (equal); investigation (equal); writing – review and editing (equal). **Thierry Lecomte:** Data curation (equal); investigation (equal); writing – review and editing (equal). **Nathalie Baize:** Data curation (equal); investigation (equal); writing – review and editing (equal). **Cedric Lecaille:** Data curation (equal); investigation (equal); writing – review and editing (equal). **Dominique Spaeth:** Data curation (equal); investigation (equal); writing – review and editing (equal). **Gael Goujon:** Data curation (equal); investigation (equal); writing – review and editing (equal). **Come Lepage:** Data curation (equal); investigation (equal); project administration (equal); writing – review and editing (equal). **David Tougeron:** Data curation (equal); investigation (equal); writing – review and editing (equal).

## FUNDING INFORMATION

This study was funded by Lilly France.

## CONFLICT OF INTEREST

None for all the authors.

## Data Availability

Data sharing is not applicable to this article as no new data were created or analyzed in this study.
